# Characteristics of stachyose-induced effects on gut microbiota and microbial metabolites *in vitro* associated with obesity in children

**DOI:** 10.3389/fnut.2024.1411374

**Published:** 2024-08-07

**Authors:** Xionge Pi, Zhi Du, Weilin Teng, Hao Fu, Lidan Hu, Jiabin Li, Jieying Ding, Xiaoxia Yang, Yinjun Zhang

**Affiliations:** ^1^Institute of Plant Protection and Microbiology, Zhejiang Academy of Agricultural Sciences, Hangzhou, China; ^2^Institute of Rural Development, Zhejiang Academy of Agricultural Sciences, Hangzhou, China; ^3^Department of Pharmacy, Children’s Hospital, Zhejiang University School of Medicine, National Clinical Research Center for Child Health, Hangzhou, China; ^4^Research Center for Clinical Pharmacy, College of Pharmaceutical Sciences, Zhejiang University, Hangzhou, China; ^5^Department of Infectious Disease Control and Prevention, HangZhou Center for Disease Control and Prevention, Hangzhou, China; ^6^Children’s Hospital, Zhejiang University School of Medicine, National Clinical Research Center for Child Health, Hangzhou, China; ^7^College of Bioengineering, Zhejiang University of Technology, Hangzhou, China

**Keywords:** gut microbiota, microbial metabolites, prebiotics, stachyose, obesity, children

## Abstract

Childhood obesity presents a serious health concern associated with gut microbiota alterations. Dietary interventions targeting the gut microbiota have emerged as promising strategies for managing obesity in children. This study aimed to elucidate the impact of stachyose (STS) supplementation on the gut microbiota composition and metabolic processes in obese children. Fecal samples were collected from 40 obese children (20 boys and 20 girls) aged between 6 and 15 and *in vitro* fermentation was conducted with or without the addition of STS, respectively, followed by 16S rRNA amplicon sequencing and analysis of short-chain fatty acids (SCFAs) and gases. Notably, our results revealed that STS supplementation led to significant alterations in gut microbiota composition, including an increase in the abundance of beneficial bacteria such as *Bifidobacterium* and *Faecalibacterium*, and a decrease in harmful bacteria including *Escherichia-Shigella*, *Parabacteroides*, *Eggerthella*, and *Flavonifractor*. Moreover, STS supplementation resulted in changes in SCFAs production, with significant increases in acetate levels and reductions in propionate and propionate, while simultaneously reducing the generation of gases such as H_2_S, H_2_, and NH_3_. The Area Under the Curve (AUC)-Random Forest algorithm and PICRUSt 2 were employed to identify valuable biomarkers and predict associations between the gut microbiota, metabolites, and metabolic pathways. The results not only contribute to the elucidation of STS’s modulatory effects on gut microbiota but also underscore its potential in shaping metabolic activities within the gastrointestinal environment. Furthermore, our study underscores the significance of personalized nutrition interventions, particularly utilizing STS supplementation, in the management of childhood obesity through targeted modulation of gut microbial ecology and metabolic function.

## 1 Introduction

Childhood obesity and overweight present significant health challenges in the pediatric population, which can extend into adulthood and increase the risk of obesity (1). Adverse health effects, such as metabolic and cardiovascular diseases, musculoskeletal problems, and psychosocial issues, can manifest early in childhood (2–4). Therefore, it is urgent to develop effective mitigation strategies.

Understanding the various factors that contribute to obesity, such as dietary patterns, genetic predispositions, sleep, mental well-being, and exercise habits, is crucial. Unhealthy dietary patterns, particularly those high in fat and fructose, significantly contribute to obesity (5–7). Research shows a decline in dietary quality from childhood to adolescence, characterized by reduced intake of fruits, vegetables, and dairy, and increased protein consumption. Low intake of dietary fiber from vegetables, fruits, and cereals notably contributes to childhood obesity (8), highlighting the need for further research into the safety and effectiveness of dietary fiber in children. The dietary choices of school-aged children and adolescents are influenced by immediate needs and are vulnerable to unhealthy food marketing. The pervasive marketing of ultra-processed foods shapes taste preferences, impacts current and future consumption, shapes brand preferences, and influences family purchasing decisions. This poses a significant risk for childhood obesity (9). During the formative years, interpersonal and social-environmental characteristics have a significant impact on dietary patterns (10, 11). Therefore, it is crucial to proactively address risk factors associated with childhood obesity during these critical stages.

The gut microbiota plays a crucial role in regulating body weight and maintaining internal environmental homeostasis (12, 13). Recent advancements in high-throughput sequencing have linked gut microbiota imbalance with the progression of overweight/obesity in children (14). There is a clear connection between the composition of gut microbiota and obesity, with differences observed between obese and lean individuals (15, 16). Stachyose (STS) is a water-soluble dietary fiber and functional oligosaccharide that has potential as a prebiotic for colonic fermentation. Previous research has shown that STS selectively fosters the growth of beneficial bacteria while inhibiting pathogenic bacteria (17–21). However, the impact of STS on gut microbiota-associated obesity in children is still limited.

The objective of this study was to investigate the modulation patterns of STS in the gut microbiota composition of obese children through *in vitro* fermentation. The study examined microbiota modulation and differences in the production of short-chain fatty acids (SCFAs) and gas. The findings provide substantial evidence of metabolic variations in STS within the gut microbiota of obese children. Further research building upon these findings will contribute to establishing STS as a dietary fiber in addressing childhood obesity.

## 2 Materials and methods

### 2.1 Reagents

Yeast extract, bile salt, L-cysteine, and heme were procured from Sigma-Aldrich (United States). Essential chemicals, including phosphate-buffered saline (PBS), NaCl, KH_2_PO_4_, K_2_HPO_4_, MgSO_4_, CaCl_2_, crotonic acid, and metaphosphoric acid, were sourced from Sangon Biotech (Shanghai) Co., Ltd., China. The YCFA medium was acquired from Ding guo chang sheng Biotechnology (Beijing) Co., Ltd., China.

### 2.2 Collection of fresh fecal samples from volunteers

The body mass index (BMI) is a fundamental metric for assessing an individual’s nutritional status. It is calculated by dividing their weight in kilograms by the square of their height in meters. BMI-for-age Z scores were calculated according to the criteria outlined by the World Health Organization and subsequently categorized based on Chinese norms (22). The study recruited 40 obese children (20 boys and 20 girls) aged 6 to 15 years based on their BMI-for-age Z scores. Exclusion criteria were implemented to ensure that none of the volunteers had a history of digestive diseases or recent treatment with antibiotics, probiotics, or prebiotics. Dietary recall interviews were conducted with these volunteers, which involved asking them to recall and describe all the foods and beverages they consumed over within the past 24 h. Multiple recalls were conducted to capture variations in dietary intake across different days of the week (23). The volunteers demonstrated a preference for highly processed and sugary foods, as outlined by the Chinese Dietary Guidelines 2016. All volunteers resided in Hangzhou, Zhejiang Province, and ethical clearance was obtained from the Ethical Committee of Hangzhou Centers for Disease Control and Prevention (No. 202047). Stool samples were promptly collected, stored at 4°C, and analyzed within a 4-h timeframe.

### 2.3 Treatment of fresh fecal samples

Fresh fecal samples, weighing 0.2 g each were carefully divided into three 1.5 mL sterile centrifuge tubes, immediately sealed, and stored at −80°C. At the same time, 0.8 g aliquots of fresh fecal samples were individually weighed and transferred to 10 mL sterile centrifuge tubes. Each tube was then filled with 8 mL of sterile phosphate-buffered saline (PBS) to ensure proper sealing. Thorough homogenization of the feces-PBS buffer mixture was achieved using a shaker, followed by careful collection of the resulting supernatant after filtration for subsequent inoculation.

### 2.4 Simulated fermentation *in vitro* of gut microbiota

The gut microbiota, extracted according to the methodology described by Liu et al., was inoculated into a simulated fermentation system as described in reference (24). The basic composition of the YCFA medium (per 100 mL) included 4.5 g/L yeast extract, 0.4 g/L bile salt, 3.0 g/L peptone, 3.0 g/L tryptone, 0.8 g/L cysteine hydrochloride, 2.5 g/L KCl, 4.5 g/L NaCl, 0.2 g/L CaCl_2_, 0.45 g/L MgCl_2_, 0.4 g/L KH_2_PO_4_, 1.0 mL Tween 80, 1.0 mL resazurin, and 2.0 mL trace element solution. To create an anaerobic environment, nitrogen was introduced after the medium was dissolved and boiled. Using a peristaltic pump (Longer Co., Ltd., China), 4.5 mL of YCFA medium was precisely injected into vials, sealed, and subjected to high-pressure steam for sterilization. In the H_Ctrl group, the gut microbiota was inoculated into the YCFA medium, which was designated as the control group. In the H_STS group, the gut microbiota was inoculated into the YCFA medium with the addition of STS at a ratio of 0.8 g per 100 mL, referred to as the treatment group.

### 2.5 Genomic DNA extraction and 16S rRNA gene sequencing

After simulated *in vitro* fermentation, distinct populations of the gut microbiota were analyzed using 16S rRNA sequencing techniques. Genomic DNA from the microbial community was extracted using the FastDNA^®^ Spin Kit for Soil (MP Biomedicals, United States) according to the manufacturer’s instructions. The V3-V4 hypervariable regions of the bacterial 16S rRNA gene were amplified using the primer pair 341F (5′-CCTAYGGGRBGCASCAG-3′) and 806R (5′-GGACTACHVGGGTWTCTAAT-3′) in a thermocycler polymerase chain reaction (PCR) system (GeneAmp 9,700, ABI, San Diego, CA, United States). PCR conditions included 3 min of pre-denaturation, followed by 27 cycles at 95°C for 30 s each, annealing at 55°C for 30 s, extension at 72°C for 45 s, and a final extension at 72°C for 10 min. Purified amplicons were pooled in equimolar amounts, and paired-end sequencing was performed on the NovaSeq PE250 platform (Illumina, San Diego, United States) based on the standard protocol of Majorbio Bio-Pharm Technology Co. Ltd. (Shanghai, China).

Upon completion of quality control processing, raw sequences were denoised using the DADA2 plugin in QIIME2 (version 2020.2) and processed to generate amplicon sequence variations (ASVs). Taxonomic assignments for ASVs were made using the SILVA 16S rRNA database (v138) and the Naive Bayes taxonomy classifier within QIIME2. All sequencing data from the raw fecal and fermentation samples have been deposited in the National Center for Biotechnology Information Short Read Archive under accession number PRJNA1040434. α-diversity evaluated at a given depth included the Ace, Chao, and Shannon indices, while β-diversity was calculated based on the Bray-Curtis distance of the ASV table using the q2-diversity plugin in QIIME2.

### 2.6 Measurement of SCFAs in simulated fermentation *in vitro*

The composition and levels of SCFAs were analyzed after 24 h of simulated *in vitro* fermentation. Six SCFAs-acetic acid (Ace), propionic acid (Pro), isobutyric acid (Isob), butyric acid (But), isovaleric acid (Isov), and pentanoic acid (Pen)-were quantified. Differences in total SCFA content and specific types of SCFAs were examined. Spearman’s rank correlation test was used to explore relationships between SCFAs and bacterial genera.

A crotonic acid/metaphosphoric acid solution was prepared by dissolving 0.6464 g crotonic acid and 2.5 g metaphosphoric acid in 100 mL deionized water. To achieve acidification within 24 h, equal volumes of fermentation broth (500 mL) and crotonic acid/metaphosphoric acid solution (100 mL) were mixed and stored at −40°C. After acidification, the samples were centrifuged at 13,000 rpm for 3 min at 4°C, and the supernatants were obtained by filtration through a 0.22 μm hydrophilic micron membrane (Millipore Express, Germany). Then, 150 μL of the filtrate was transferred to a test tube.

After loading the sample solution into the gas chromatograph (GC-2010 Plus, Shimadzu, Japan), the aging procedure was started. The column temperature was started at 80°C for 1 min, increased to 190°C at a rate of 10°C/min, held for 0.50 min, further increased to 240°C at 40°C/min, and held for 5 min. The flame ionization detector (FID) was set at 240°C, the gasification chamber was set at 240°C, and the carrier gas consisted of a nitrogen flow rate of 20 mL/min, a hydrogen flow rate of 40 mL/min, and an air flow rate of 400 mL/min. Data acquisition and processing were performed using LabSolutions software (Shimadzu, Japan).

### 2.7 Measurement of gas in simulated fermentation *in vitro*

Gas production from bottles with completed fermentation after 24 h was evaluated using a fermentation gas analyzer equipped with five sensitive gas transducers (Empaer, China) operating at a constant room temperature of 25°C, according to the protocol described by Ye et al. (25). After activation of the gas detector and completion of preheating, the record button was pressed, and the inlet and outlet ports were connected to the syringe bottle via a rubber tubing and a disposable syringe needle, respectively. Careful measures were taken to prevent water from entering the apparatus by ensuring that the needle remained clear of the liquid surface of the culture medium during measurements. Peak concentrations of the five gases-methane (CH_4_), hydrogen sulfide (H_2_S), ammonia (NH_3_), carbon dioxide (CO_2_), and hydrogen (H_2_)-were carefully recorded. After all five gas levels were reduced to zero, the analysis process was repeated for the next sample bottle.

### 2.8 Data analysis

Data are presented as mean ± standard error of the mean and reflect the aggregated results of all independent experiments, each performed in triplicate. Statistical analyses and graphs involving gases, short-chain fatty acids (SCFAs), and bacterial genera were performed using SPSS 23.0 (IBM Corp., United States) and GraphPad Prism 8.0.1 (GraphPad Software, United States). Normality of data distribution was assessed using the Shapiro–Wilk test. In cases of normal distribution, a paired t-test was used; otherwise, a paired Wilcoxon rank-sum test was used for pre-and post-fermentation comparisons. Additionally, the Wilcoxon rank sum test was used to compare α-diversity between the two groups. β-diversity, indicating structural shifts in the microbial community at the genus level, was analyzed using Principal Component Analysis (PCA). The LEfSe analysis identified the microbial taxa that exhibited differential abundance, applying a threshold of an LDA score greater than 3.0. The Area Under the Curve (AUC)-Random Forest algorithm was employed for the purpose of ranking the relative importance of the bacteria and metabolites, and for model validation. Redundancy analysis (RDA) was employed to evaluate the interrelationship between samples and fermented metabolites (SCFAs and gases). Predictive functional analysis was performed using PICRUSt 2. Microbiota data were analyzed on the Majorbio Cloud Platform.^1^

## 3 Results

### 3.1 Diversity analysis

**Figure 1:**
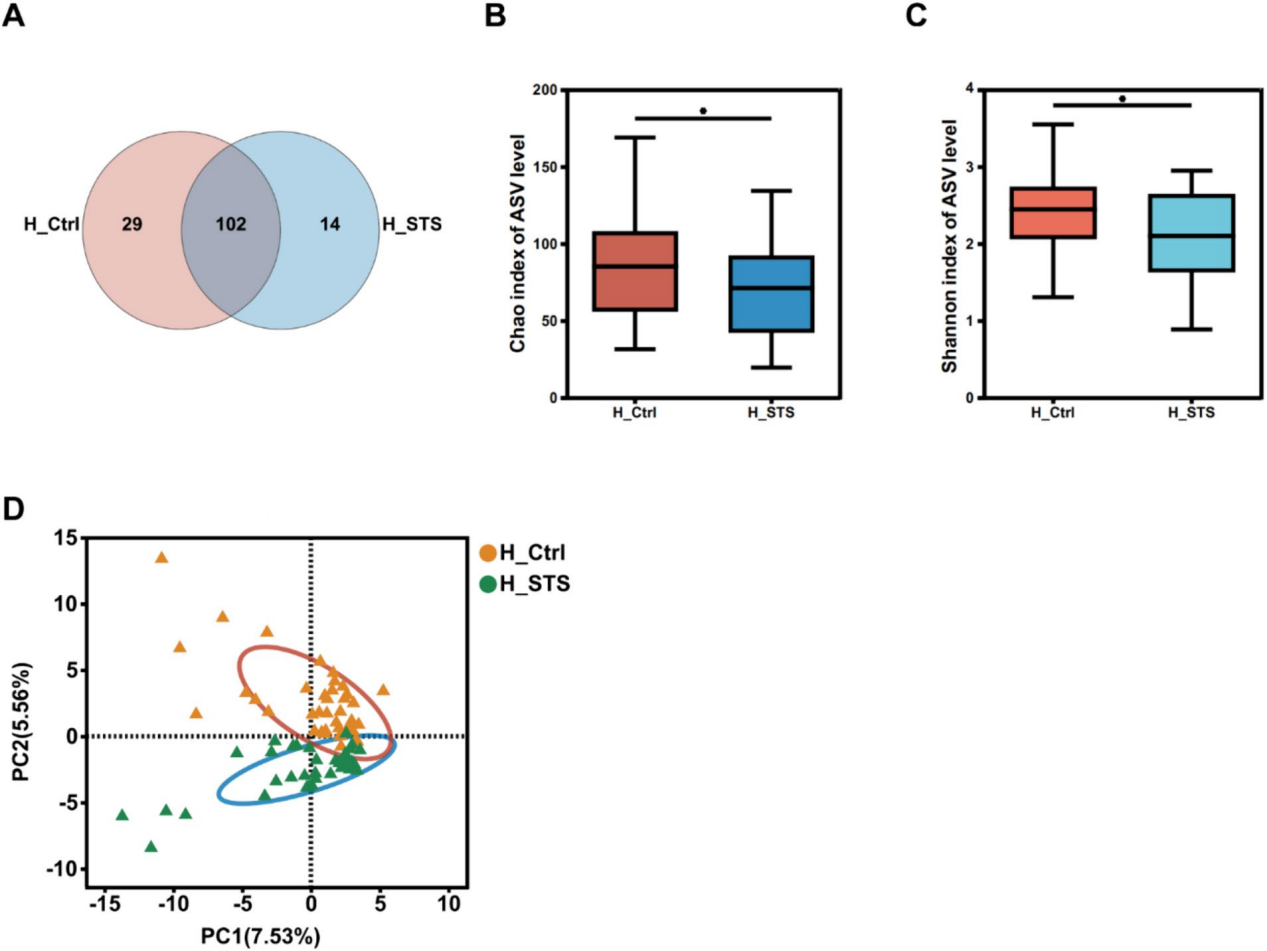
Diversity of gut microbiota. **(A)** The Venn diagram, based on ASV levels, illustrates the number of genera in the H_Ctrl (orange) and H_STS (blue) groups, as well as the common genera (purple) between the two groups. α-diversity in the H_Ctrl and H_STS groups, assessed using the **(B)** Chao index (*p* = 0.03766) and **(C)** Shannon index (*p* = 0.03075), is displayed with significance indicated by asterisks (*0.01 < *p* ≤ 0.05; **0.001 < *p* ≤ 0.01; ***0.0001 < *p* ≤ 0.001; ****p ≤ 0.0001). **(D)** β-diversity at the genus level is visualized through PCA (*p* = 0.001) comparing the H_Ctrl and H_STS groups.

After 24 h of *in vitro* fermentation, the gut microbiota was analyzed by 16S rRNA sequencing. A Venn diagram was used to provide a comprehensive representation of the gut microbial profile. Comparison of the microbial profiles between the H_Ctrl and H_STS groups revealed 102 common genera (Figure 1A). Figures 1B,C show the α-diversity in both groups. Both the Chao (*p* = 0.03766) and Shannon (*p* = 0.03075) indices showed statistically significant differences between the two groups (*p* < 0.05), indicating remarkable diversity differences between the H_Ctrl and H_STS groups. Figure 1D illustrate the β-diversity of the H_Ctrl and H_STS groups. The PCA (*p* = 0.001) revealed significant differences in the bacterial community structure at the genus level between the H_Ctrl and H_STS groups during *in vitro* fermentation (Figure 1D).

### 3.2 The compositions of microbiota

**Figure 2:**
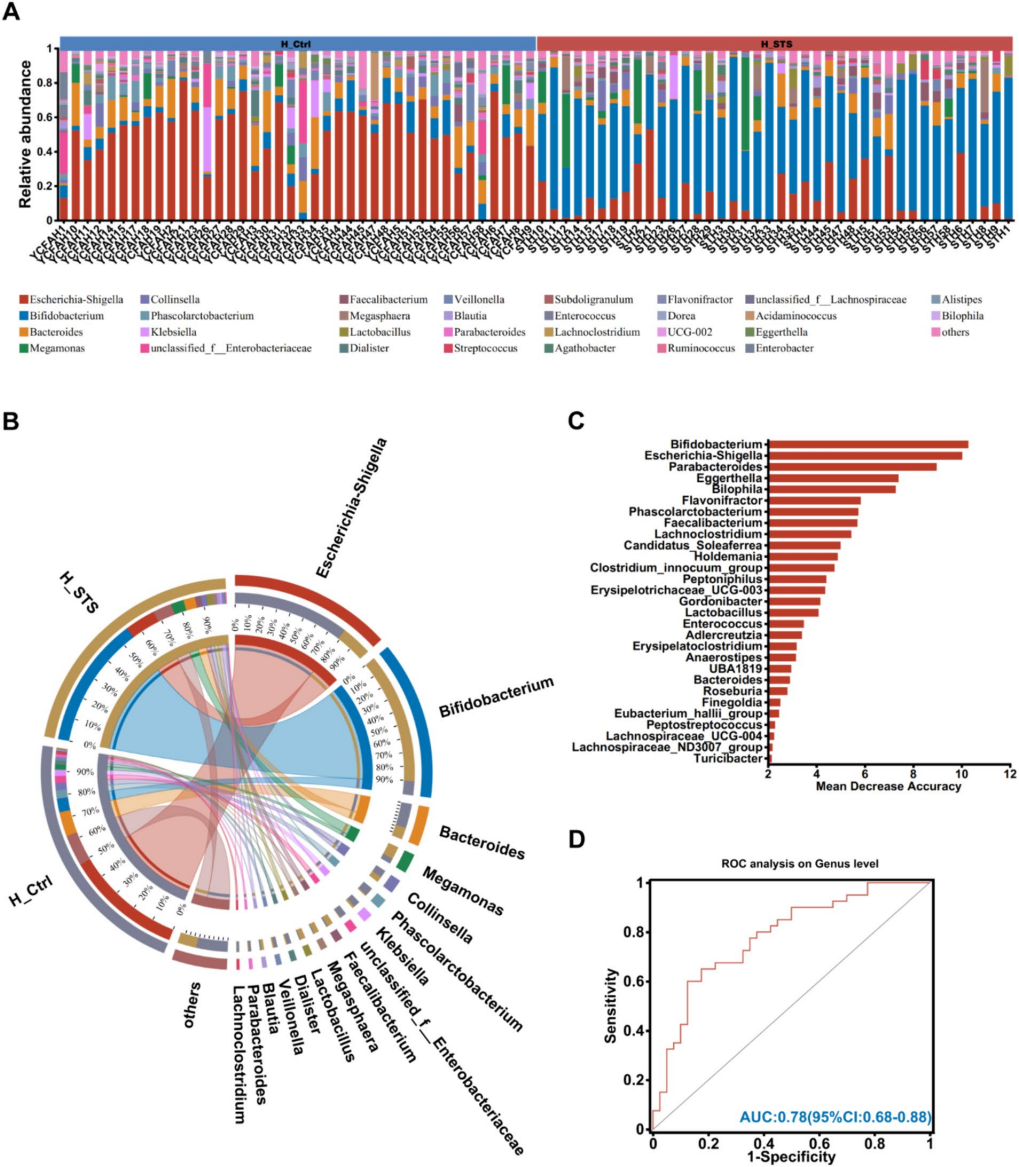
Composition of gut microbiota. **(A)** The community bar plot analysis at the genus level visually represents the relative abundance of gut microbiota in individual samples from the H_Ctrl and H_STS groups. **(B)** Circos analysis at the genus level offers a comprehensive view of the abundance relationship between samples and bacterial communities. **(C)** The bar plot illustrates the variable importance of gut microbiota at the genus level, constructed through random forest. **(D)** The performance of the model candidates is evaluated using ROC analysis of gut microbiota at the genus level, with AUC values indicating diagnostic accuracy. AUC ≤ 0.5 signifies no diagnostic value, AUC = 0.5 ~ 0.7 indicates low accuracy, AUC = 0.7 ~ 0.9 suggests a certain degree of accuracy, and AUC ≥ 0.9 indicates high accuracy.

The bar plots in Figure 2A show the relative abundance of different bacterial genera in the 80 fecal samples. Complementing these results, the Circos analysis visually illustrates the relative abundance relationships between bacterial communities at the genus and group level, as shown in Figure 2B. Significant differences were observed in genera such as *Bifidobacterium* (6.15% vs. 55.08%), *Escherichia-Shigella* (49.11% vs. 13.21%), *Parabacteroides* (1.06% vs. 0.24%), *Eggerthella* (0.64% vs. 0.03%), *Flavonifractor* (0.78% vs. 0.05%), *Bilophila* (0.52% vs. 0.06%), *Phascolarctobacterium* (3.51% vs. 0.51%), *Faecalibacterium* (0.49% vs. 2.56%), and *Lachnoclostridium* (1.00% vs. 0.05%). To identify potential gut microbiota biomarkers, the AUC-Random Forest algorithm was employed and the optimal model was identified that maximized the AUC value of the Receiver Operating Characteristic (ROC) curve. In the validation cohorts of the H_Ctrl and H_STS groups, we selected the top 30 bacterial genera were shown in Figure 2C. Sensitivity and specificity analyses were conducted for the top 80 bacterial genera at the maximum AUC value from the random forest algorithm, resulting in the AUC of 0.78 (95% confidence interval [CI]: 0.68–0.88), as depicted in Figure 2D.

### 3.3 The differences of microbiota

**Figure 3:**
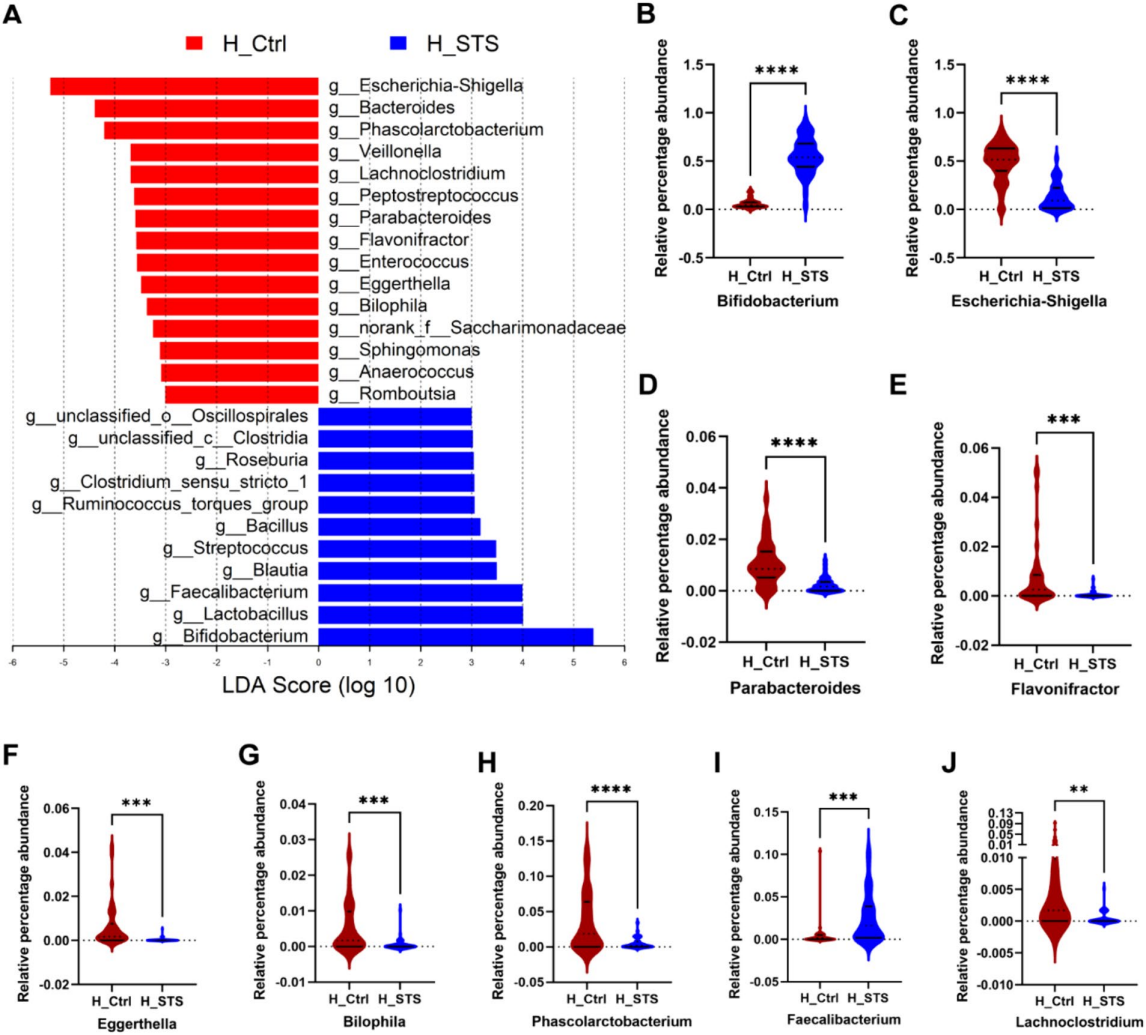
Difference of gut microbiota. **(A)** Gut microbiota comparisons at the genus level between the H_Ctrl and H_STS groups are examined using LEfSe (LDA > 3, *p* < 0.05). **(B–J)** The relative percentage abundance differences of the top 9 variables, importance of gut microbiota at the genus level in Figure 2C, are illustrated. Significance thresholds are denoted by asterisks (*0.01 < *p* ≤ 0.05; **0.001 < *p* ≤ 0.01; ***0.0001 < *p* ≤ 0.001; ****p ≤ 0.0001).

To assess potential differences in bacterial genus enrichment between the H_Ctrl and H_STS groups, we performed LEfSe analysis using Linear Discriminant Analysis (LDA) effect size. As shown in Figure 3A, 26 bacterial genera showed disparities between the two groups. Notably, the significant enrichment of 15 bacterial genera in the H_Ctrl group, such as *Escherichia-Shigella*, *Parabacteroides*, *Eggerthella*, *Flavonifractor*, *Bilophila*, *Phascolarctobacterium*, and *Lachnoclostridium* (Figures 3C–H,J). Conversely, the H_STS group manifested a higher abundance of 11 bacterial genera, including *Bifidobacterium* and *Faecalibacterium* (Figures 3B,I).

### 3.4 SCFAs analysis

**Figure 4:**
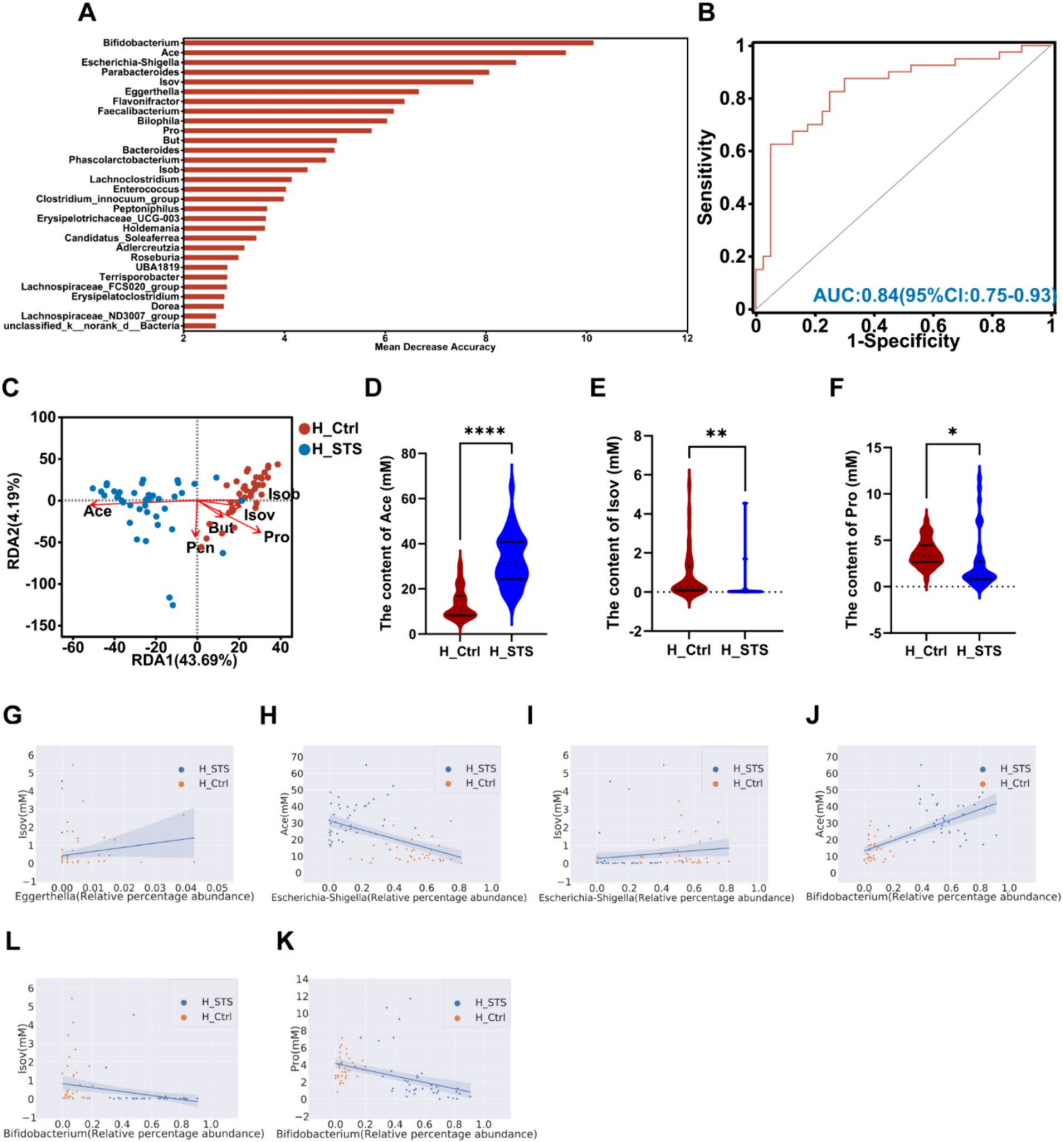
SCFAs levels in the H_Ctrl and H_STS groups. **(A)** The bar plot displays the variable importance of gut microbiota at the genus level and SCFAs determined through random forest. **(B)** The ROC analysis evaluates the performance of model candidates based on gut microbiota at the genus level and SCFAs, with AUC values indicating diagnostic accuracy. AUC ≤ 0.5 indicates no diagnostic value, AUC = 0.5 ~ 0.7 indicates low accuracy, AUC = 0.7 ~ 0.9 indicates a certain degree of accuracy, and AUC ≥ 0.9 indicates high accuracy. **(C)** RDA analysis of SCFAs and samples in the H_Ctrl and H_STS groups. **(D–F)** Significant differences in SCFAs levels between the H_Ctrl and H_STS groups are shown, with statistical significance thresholds indicated by asterisks (*0.01 < *p* ≤ 0.05; **0.001 < *p* ≤ 0.01; ***0.0001 < *p* ≤ 0.001; ****p ≤ 0.0001). Scatterplots illustrate the significant correlations (cor ≥ 0.5) between SCFAs and gut microbiota. The correlations are as follows: **(G)** cor = 0.506, *p* = 1.69e-06; **(H)** cor = −0.5315, *p* = 3.93e-07; **(I)** cor = 0.5129, *p* = 1.15e-06; **(J)** cor = 0.696, *p* = 7.69e-13; **(K)** cor = −0.6097, *p* = 1.93e-09; **(L)** cor = −0.5476, *p* = 1.47e-07.

After 24 h of *in vitro* fermentation, we analyzed the composition and content of SCFAs, including six types: Ace, Pro, Isob, But, Isov, and Pen. To evaluate the potential of gut microbiota and SCFAs as biomarkers, we performed the AUC-Random Forest algorithm, and selected the top 30 features (SCFAs and bacterial genera) were shown in Figure 4A. Sensitivity and specificity analyses for the top 100 features at the maximum AUC value from the random forest algorithm revealed the AUC of 0.84 (95% confidence interval [CI]: 0.75–0.93; Figure 4B). In addition, the RDA was used to explore the relationship between SCFAs and samples. The RDA plot shows the distribution of samples in the H_Ctrl and H_STS groups. Ace showed a positive association with samples in the H_STS group and a negative association with samples in the H_Ctrl group. Conversely, Isov and Pro showed a positive association with samples in the H_Ctrl group and a negative association with samples in the H_STS group (Figure 4C). Compared to the H_Ctrl group, the H_STS group had significantly higher levels of Ace (*p* ≤ 0.0001) and significantly lower levels of Isov (0.001 < *p* ≤ 0.01) and Pro (0.01 < *p* ≤ 0.05; Figures 4D–F). Furthermore, scatter plots and Spearman correlation coefficients were used to analyze the association and significance between SCFAs and bacterial genera (Figures 4G–L). *Eggerthella* showed a significant positive correlation (cor > 0.5, *p* < 0.05) with Isov. *Escherichia-Shigella* showed a significant positive correlation (cor > 0.5, *p* < 0.05) with Isov and Pro, but a negative correlation (cor < −0.5, *p* < 0.05) with Ace. *Bifidobacterium* showed a significant positive correlation (cor > 0.5, *p* < 0.05) with Ace, but a significant negative correlation (cor < −0.5, *p* < 0.05) with Isov and Pro.

### 3.5 Gas analysis

**Figure 5:**
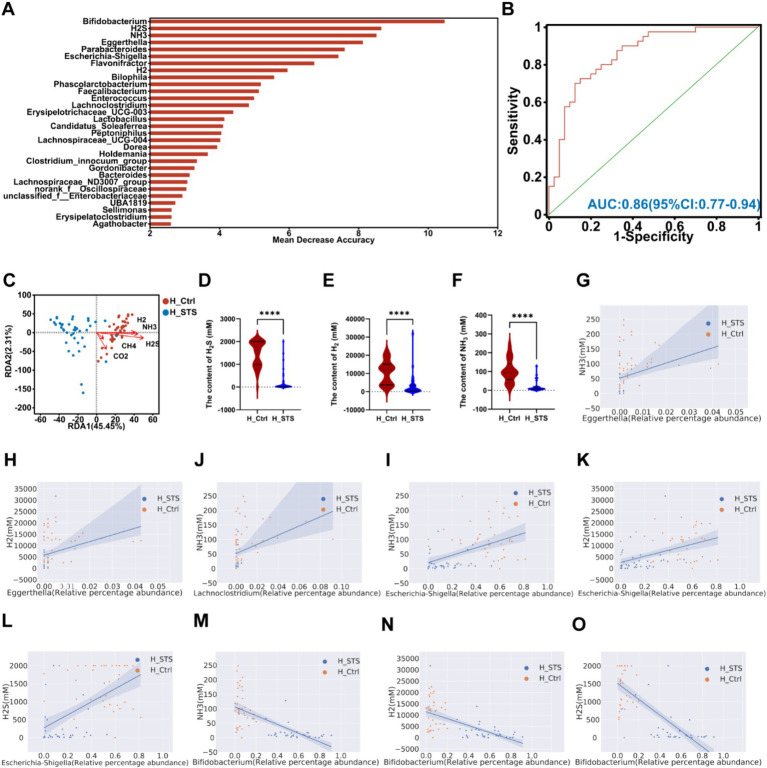
Content of gas in different media. **(A)** The bar plot depicts the variable importance of gut microbiota at the genus level and gases as determined by random forest. **(B)** The ROC analysis assesses the performance of model candidates based on gut microbiota at the genus level and gases, with AUC values indicating diagnostic accuracy. AUC ≤ 0.5 indicates no diagnostic value, AUC = 0.5 ~ 0.7 indicates low accuracy, AUC = 0.7 ~ 0.9 indicates a certain degree of accuracy, and AUC ≥ 0.9 indicates high accuracy. **(C)** RDA analysis of gases and samples in the H_Ctrl and H_STS groups. **(D–F)** Significant differences in gas levels between the H_Ctrl and H_STS groups are presented, with statistical significance thresholds indicated by asterisks (*0.01 < *p* ≤ 0.05; **0.001 < *p* ≤ 0.01; ***0.0001 < *p* ≤ 0.001; ****p ≤ 0.0001). Scatterplots illustrate the significant correlations (cor ≥ 0.5) between gases and gut microbiota. The correlations are as follows: **(G)** cor = 0.545, *p* = 1.73e-07; **(H)** cor = 0.5299, *p* = 4.31e-07; **(I)** cor = 0.5221, *p* = 6.82e-07; **(J)** cor = 0.554, *p* = 9.76e-08; **(K)** cor = 0.5413, *p* = 2.17e-07; **(L)** cor = 0.5621, *p* = 5.77e-08; **(M)** cor = −0.7368, *p* = 6.66e-15; **(N)** cor = −0.6259, *p* = 5.37e-10; **(O)** cor = −0.7229, *p* = 3.69e-14.

After 24 h of *in vitro* fermentation, we analyzed the composition and concentration of gases, including five types: CH_4_, H_2_S, NH_3_, CO_2_, and H_2_. To evaluate the potential of gut microbiota and gases as biomarkers, the AUC-Random Forest algorithm was performed. The top 30 features (gases and bacterial genera) are presented in Figure 5A. Sensitivity and specificity analyses using the ROC curve for the top 46 features at the maximum AUC value from the random forest algorithm revealed the AUC of 0.86 (95% confidence interval [CI]: 0.77–0.94; Figure 5B). In addition, we used the RDA to explore the relationship between gases and samples. The RDA plot shows the sample distribution in the H_Ctrl and H_STS groups. All gases showed a negative association with samples in the H_STS group and a positive association with samples in the H_Ctrl group (Figure 5C). In contrast to the H_Ctrl group, the H_STS group showed significantly lower levels of H_2_S (*p* ≤ 0.0001), H_2_ (*p* ≤ 0.0001), and NH_3_ (*p* ≤ 0.0001; Figures 5D–F). In addition, scatter plots and Spearman correlation coefficients were used to examine the association and significance between gases and bacterial genera (Figures 5G–O). Notably, *Eggerthella* showed a significant positive correlation (cor > 0.5, *p* < 0.05) with NH_3_ and H_2_, while *Lachnoclostridium* showed a positive association (cor > 0.5, *p* < 0.05) with NH_3_. *Escherichia-Shigella* showed a significant positive correlation (cor > 0.5, *p* < 0.05) with NH_3_, H_2_ and H_2_S. Conversely, *Bifidobacterium* showed a significant negative correlation (cor < −0.5, *p* < 0.05) with NH_3_, H_2_ and H_2_S.

### 3.6 Functional prediction

**Figure 6:**
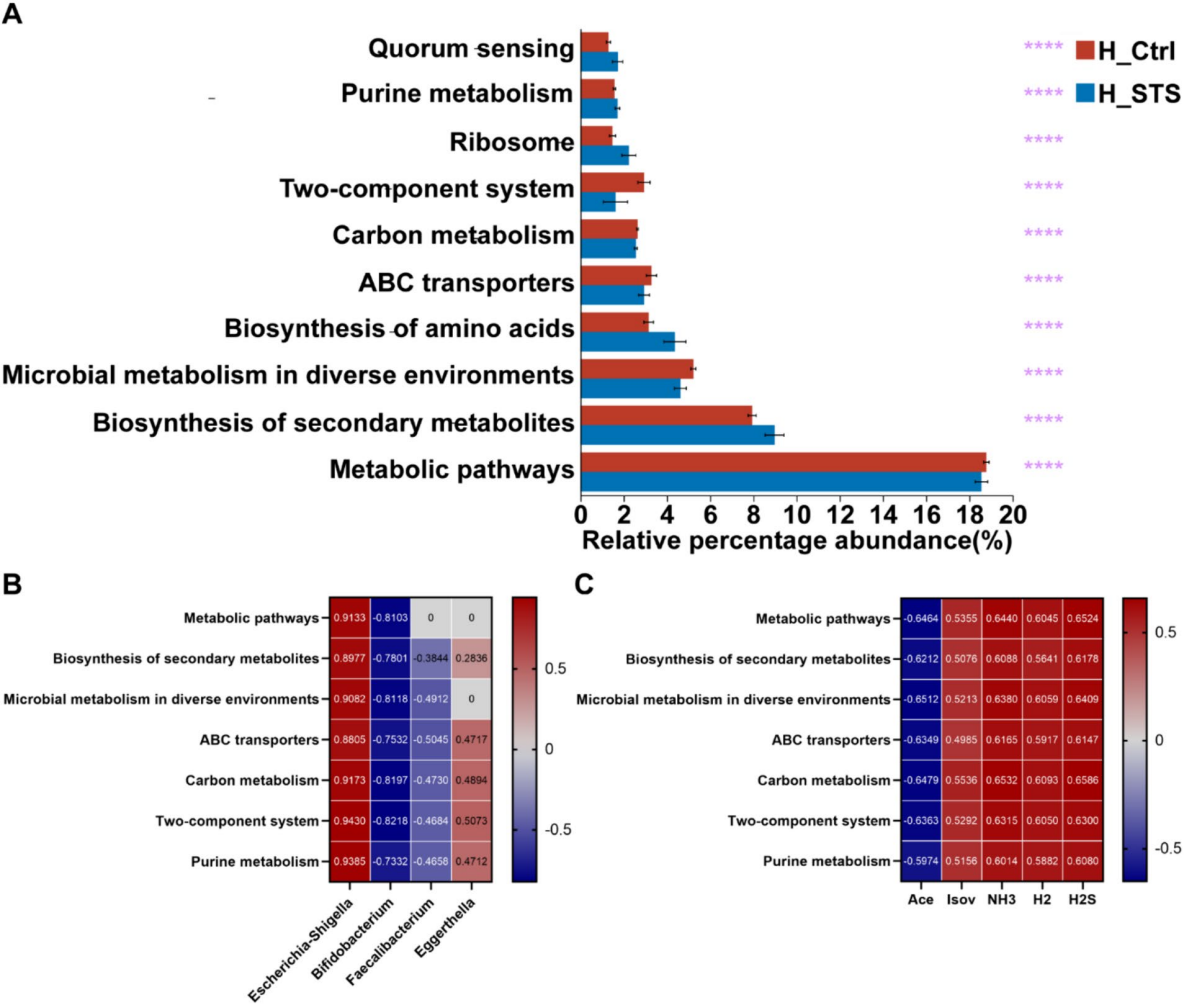
PICRUSt 2 analysis and Wilcoxon rank-sum test bar plots. **(A)** The top 10 metabolic pathways at Level 3, based on KEGG categories, are presented with statistical significance thresholds indicated by asterisks (*0.01 < *p* ≤ 0.05; **0.001 < *p* ≤ 0.01; ***0.0001 < *p* ≤ 0.001; ****p ≤ 0.0001). **(B,C)** Depict correlation analyses between metabolic pathways and the top 7 relative abundances of gut microbiota at the genus level.

To predict and compare the functional capacities of the gut microbiota between the H_Ctrl and H_STS groups, we performed PICRUSt 2 analysis using the Kyoto Encyclopedia of Genes and Genomes (KEGG) database. At level 3, we visualized the top 10 pathways with notable differences between the H_Ctrl and H_STS groups by bar chart analysis (Figure 6A). Among these pathways, five showed significantly higher enrichment in the H_STS group, including biosynthesis of secondary metabolites (*p* ≤ 0.0001), biosynthesis of amino acids (*p* ≤ 0.0001), ribosome (*p* ≤ 0.0001), quorum sensing (*p* ≤ 0.0001), and purine metabolism (*p* ≤ 0.0001). Concurrently, five pathways were notably more enriched in the H_Ctrl group, encompassing metabolic pathways (*p* ≤ 0.0001), microbial metabolism in diverse environments (*p* ≤ 0.0001), ABC transporters (*p* ≤ 0.0001), carbon metabolism (*p* ≤ 0.0001), and Two-component system (*p* ≤ 0.0001). Predicted pathways, including metabolic pathways, biosynthesis of secondary metabolites, microbial metabolism in diverse environments, ABC transporters, carbon metabolism, two-component systems, and purine metabolism, showed the highest positive correlation (cor > 0.5) with Escherichia-Shigella and the highest negative correlation (cor < −0.5) with Bifidobacterium in the microbial community (Figure 6B). This set of pathways also showed a significant negative correlation with Ace and a significant positive correlation with Isov, NH_3_, H_2_, and H_2_S (Figure 6C).

## 4 Discussion

Unhealthy dietary patterns, especially those with low dietary fiber intake, are widely recognized as significant contributors to gut microbiota imbalance in obese children. This study utilized simulated *in vitro* fermentation to characterize the gut microbiota, sequenced the microbial community using 16S rRNA amplicon analysis, and quantified SCFAs and gases produced by the microbiota. We investigated the effects of STS as a dietary fiber on the gut microbiota and its metabolites in obese children using these methods.

The human gastrointestinal tract hosts a vast array of microorganisms, which significantly influence human health (26). The intricate interplay between the metabolism of the gut microbiota and the physiological stability of the host has profound implications for health (27, 28). Imbalances in the composition of the gut microbiota can contribute to various diseases that impact human health, including obesity and type 2 diabetes mellitus, both of which are closely associated with alterations in the gut microbiota (29, 30). Therefore, investigating the gut microbiota is crucial for understanding human gut health. Previous studies have highlighted the prebiotic properties of STS, underscoring its effectiveness in modulating the equilibrium of the gut microbiota. This modulation leads to structural changes in microbial communities, resulting in an increase in beneficial probiotics such as *Bifidobacterium* and *Lactobacillus*, while concurrently inhibiting the growth of harmful bacteria like *Clostridium perfringens* (17). These collective actions confer significant advantages on the overall health of the host.

In this study, after 24 h of *in vitro* fermentation, STS demonstrated the capacity to influence the composition of the gut microbiota (Figure 2). To gain insight into the mechanisms underlying alterations in gut microbiota composition in childhood obesity, it is essential to examine the influence of dietary habits and microbial composition on the microbial ecosystem. In this study, we conducted a comprehensive analysis of the significant enrichment of specific bacterial genera in the control group (H_Ctrl) and the STS supplementation group (H_STS; Figure 3). In the H_Ctrl group, there was a notable enrichment in potentially pathogenic and opportunistic bacterial genera such as *Escherichia-Shigella*, *Eggerthella*, and *Bilophila*, which can produce harmful metabolites and trigger immune responses, contributing to the metabolic disturbances observed in childhood obesity (31–34). The presence of bacterial genera involved in proteolytic fermentation, such as *Parabacteroides* and *Flavonifractor*, suggests a diet high in protein and low in fiber, which further exacerbates metabolic imbalance and promotes an environment conducive to obesity (35, 36). Conversely, the higher abundance of beneficial bacterial genera such as *Bifidobacterium* and *Faecalibacterium* in the H_STS group is indicative of the positive impact of STS supplementation. Consequently, the inhibition of harmful bacterial genera and the enrichment of beneficial bacterial genera through STS supplementation can help to mitigate the adverse effects of obesity by promoting a healthier gut microbiota.

SCFAs such as Ace, Pro, But, Isob, Isov, and Pen are critical metabolites produced by gut microbiota during the fermentation of dietary fibers. SCFAs play a pivotal role in maintaining gut health, regulating metabolism, and influencing immune function (37, 38). The analysis of changes in SCFAs levels and their correlation with specific bacterial genera in the control (H_Ctrl) and STS supplementation (H_STS) groups provides insights into the metabolic and microbial shifts associated with STS supplementation in childhood obesity (Figure 3). In the H_Ctrl group, higher levels of Pro and Isov were observed in positive correlations *Escherichia-Shigella*. While Pro has beneficial effects, such as regulating glucose metabolism and appetite, high level of Pro in a dysbiotic gut may reflect an imbalance contributing to metabolic disturbances (39, 40). Isov, a product of branched-chain amino acid (BCAA) fermentation, is indicative of elevated protein fermentation activity (41). High level of Isov is often associated with diets high in protein and low in fiber (42). The association of *Escherichia-Shigella* with elevated levels of these SCFAs may contribute to increased energy extraction from the diet and altered fat storage, thereby potentially contributing to the development of childhood obesity. Conversely, the H_STS group, which exhibited higher level of Ace and positive correlations with beneficial bacteria such as *Bifidobacterium*. Ace is an essential energy source, influences lipid metabolism, and has anti-inflammatory properties (43). *Bifidobacterium* is known for its beneficial effects on gut health, including enhancing gut barrier function, modulating immune responses, and inhibiting pathogenic bacteria (44). Elevated level of Ace, driven by *Bifidobacterium*, have the potential to contribute to an increased energy harvest from the diet and to influence pathways related to facilitating glycolysis and energy expenditure (45). It is possible that this may have complex effects on obesity. The random forest analysis identified key features (SCFAs and bacterial genera) that differentiate between the H_Ctrl and H_STS groups, achieving a maximum AUC of 0.84. This demonstrates the effectiveness of SCFA profiles and microbiota composition as biomarkers for distinguishing these groups. The RDA plots revealed distinct associations between specific SCFAs and the microbiota compositions of each group. Ace was positively associated with the H_STS group, while Isov and Pro were positively associated with the H_Ctrl group. These resullts underscore the impact of STS supplementation on promoting a healthier SCFA profile.

Gases produced by the gut microbiota, CH_4_, H_2_S, NH_3_, CO_2_, H_2_, serve as key indicators of microbial activity and gut health (46). The investigation of alterations in gas production in the context of childhood obesity and the assessment of the impact of STS supplementation, can provide valuable insights into the dynamics of the gut microbiota and their influence on metabolic health. The H_STS group exhibited significantly lower level of H_2_S, H_2_, and NH_3_ compared to the H_Ctrl group. H_2_S, a byproduct of sulfate-reducing bacteria, has been linked to the damaging of the gut lining, which can contribute to the development of inflammation and gastrointestinal disorders (47). A significant positive correlation was observed between *Escherichia-Shigella* and H_2_S, indicating that this pathogenic genus thrives in and contributes to a low in dieray fiber with high H_2_S production, which is harmful to gut health (48, 49). H_2_ is a byproduct of microbial fermentation of carbohydrates and fibers. Although H_2_ itself is not harmful, its accumulation affects other microbial processes and can indicate high fermentative activity (49). Positive correlations with *Eggerthella* and *Escherichia-Shigella* suggest that these bacterial genera contribute to higher H_2_ production, reflecting a gut environment with increased fermentation associated with dysbiosis and metabolic imbalance (50, 51). NH_3_ is produced during protein fermentation, and elevated level indicates increased proteolytic activity, which is often linked to gut dysbiosis and inflammation (52). Positive correlations with *Eggerthella*, *Lachnoclostridium*, and *Escherichia-Shigella* highlight these genera’s role in proteolytic fermentation, which produces NH_3_. These bacterial genera are involved in various metabolic activities, including protein fermentation and the production of metabolites that affect energy balance and fat storage. Their association with increased NH_3_ level suggests they may significantly impact the gut microbiome’s role in childhood obesity by altering the gut environment and metabolic functions (53–55). These findings indicate that STS supplementation results in a reduction in gas production, which is indicative of a healthier gut microbiota composition.

Furthermore, after STS supplementation, the analysis using PICRUSt 2 analysis and heatmap revealed seven metabolic pathways with significant negative correlations with *Bifidobacterium* and Ace, while showing significant positive correlations with *Escherichia-Shiguela*, H_2_S, and NH_3_ (Figure 6). These findings provide preliminary evidence that STS supplementation may influence the gut microbiota and its metabolic output in obese children. However, these findings are based on correlations and require further validation through additional studies. Future research should aim to confirm these associations and elucidate the causal relationships between STS treatment, changes in gut microbiota, and their metabolic pathways. This could lead to the development of novel therapeutic strategies targeting the gut microbiota for the management of obesity in children.

It is crucial to address the notable limitations in the study. Firstly, the use of fecal extracts of gut microbiota may not fully represent the entire spectrum of gut microbiota due to potential discrepancies in composition compared to the gastrointestinal tract. Secondly, relying on *in vitro* models, although beneficial for controlled experiments, lacks the complexity of human physiology and may not capture all dynamic interactions present in the body. Lastly, the microbiota analysis in the study was based on taxonomic profiles from 16S rRNA gene sequencing, which offers a broad overview but may lack detailed insights compared to more advanced methods like complete shotgun metagenome sequencing. These limitations should be considered when interpreting the results, and future research could explore advanced techniques and *in vivo* models to enhance understanding.

## 5 Conclusion

Childhood obesity represents a substantial health hazard, with disrupted dietary patterns playing a pivotal role in its development, underscoring the urgency of implementing early dietary interventions to mitigate associated health concerns. The results of this study lay the groundwork for investigating the impact of diet, specifically STS, on the gut microbiota and provide insights into the potential development of personalized nutrition programs. STS emerges not only as a factor shaping the composition of the gut microbial community, but also as a significant influencer of microbial metabolites. The integration of various analyses, including random forest, RDA, scatterplot, and PICRUSt 2, facilitates the identification of correlations between gut microbiota, metabolites, and pathways. STS has the potential to play a pivotal role in addressing childhood obesity by delineating traits that modulate gut microbiota metabolic output and thereby promote gut health. The ability of STS to regulate key microbial metabolites associated with childhood obesity positions it as a valuable therapeutic strategy in the field of personalized medicine. The exploration of dietary interventions, particularly those involving STS, may pave the way for effective strategies aimed at optimizing the metabolic profile of the gut microbiota in the context of childhood obesity.

## Data availability statement

The data presented in the study are deposited in the NCBI Sequence Read Archive repository, accession number PRJNA1040434.

## Ethics statement

The study was conducted in accordance with the Declaration of Helsinki and approved by the Ethics Committee of Hangzhou Centers for Disease Control and Prevention (No. 20047) for studies involving humans. The studies were conducted in accordance with the local legislation and institutional requirements. Written informed consent for participation in this study was provided by the participants’ legal guardians/next of kin.

## Author contributions

XP: Data curation, Funding acquisition, Writing – original draft, Formal analysis. ZD: Data curation, Formal analysis, Writing – review & editing, Methodology. WT: Writing – review & editing. HF: Writing – review & editing. LH: Writing – review & editing. JL: Writing – review & editing. JD: Writing – review & editing. XY: Writing – review & editing. YZ: Supervision, Methodology, Project administration, Writing – review & editing.

## Footnotes

^1^www.majorbio.com

## References

[1] Jebeile H Kelly AS O'Malley G Baur LA. Obesity in children and adolescents: epidemiology, causes, assessment, and management. Lancet Diabetes Endocrinol. (2022) 10:351–65. doi: 10.1016/S2213-8587(22)00047-X, PMID: 35248172 PMC9831747

[2] Turta O Rautava S. Antibiotics, obesity and the link to microbes-what are we doing to our children? BMC Med. (2016) 14:57. doi: 10.1186/s12916-016-0605-7, PMID: 27090219 PMC4836077

[3] Baker JL Olsen LW Sorensen TI. Childhood body-mass index and the risk of coronary heart disease in adulthood. N Engl J Med. (2007) 357:2329–37. doi: 10.1056/NEJMoa072515, PMID: 18057335 PMC3062903

[4] Juonala M Magnussen CG Berenson GS Venn A Burns TL Sabin MA. Childhood adiposity, adult adiposity, and cardiovascular risk factors. N Engl J Med. (2011) 365:1876–85. doi: 10.1056/NEJMoa1010112 22087679

[5] Zhang Y Zhang X Li J Zhong H Pan CW. Associations of outdoor activity and screen time with adiposity: findings from rural Chinese adolescents with relatively low adiposity risks. BMC Public Health. (2020) 20:1769. doi: 10.1186/s12889-020-09897-7, PMID: 33228624 PMC7684968

[6] Zhang C Yin A Li H Wang R Wu G Shen J. Dietary modulation of gut microbiota contributes to alleviation of both genetic and simple obesity in children. EBioMedicine. (2015) 2:968–84. doi: 10.1016/j.ebiom.2015.07.007, PMID: 26425705 PMC4563136

[7] Zhou P Li R Liu K. The neighborhood food environment and the onset of child-Hood obesity: a retrospective time-trend study in a mid-Sized City in China. Front Public Health. (2021) 9:688767. doi: 10.3389/fpubh.2021.688767, PMID: 34381750 PMC8350029

[8] Taverno Ross SE Militello G Dowda M Pate RR. Changes in diet quality in youth living in South Carolina from fifth to 11th grade. J Nutr Educ Behav. (2020) 52:928–34. doi: 10.1016/j.jneb.2020.03.001 32334976 PMC7554150

[9] Neri D Steele EM Khandpur N Cediel G Zapata ME Rauber F. Ultraprocessed food consumption and dietary nutrient profiles associated with obesity: a multicountry study of children and adolescents. Obes Rev. (2022) 23:e13387. doi: 10.1111/obr.13387, PMID: 34889015

[10] Toumpakari Z Haase AM Johnson L. Adolescents' non-Core food intake: a description of what, where and with whom adolescents consume non-Core foods. Public Health Nutr. (2016) 19:1645–53. doi: 10.1017/S1368980016000124, PMID: 26878965 PMC10270939

[11] Ambrosini GL Oddy WH Robinson M O'Sullivan TA Hands BP de Klerk NH. Adolescent dietary patterns are associated with lifestyle and family psycho-social factors. Public Health Nutr. (2009) 12:1807–15. doi: 10.1017/S1368980008004618, PMID: 19161648

[12] Arumugam M Raes J Pelletier E Le Paslier D Yamada T Mende DR. Enterotypes of the human gut microbiome. Nature. (2011) 473:174–80. doi: 10.1038/nature09944, PMID: 21508958 PMC3728647

[13] Gomaa EZ. Human gut microbiota/microbiome in health and diseases: a review. Antonie Van Leeuwenhoek. (2020) 113:2019–40. doi: 10.1007/s10482-020-01474-7, PMID: 33136284

[14] Riva A Borgo F Lassandro C Verduci E Morace G Borghi E. Pediatric obesity is associated with an altered gut microbiota and discordant shifts in Firmicutes populations. Environ Microbiol. (2017) 19:95–105. doi: 10.1111/1462-2920.13463, PMID: 27450202 PMC5516186

[15] Ley RE Turnbaugh PJ Klein S Gordon JI. Microbial ecology: human gut microbes associated with obesity. Nature. (2006) 444:1022–3. doi: 10.1038/4441022a 17183309

[16] Turnbaugh PJ Hamady M Yatsunenko T Cantarel BL Duncan A Ley RE. A Core gut microbiome in obese and lean twins. Nature. (2009) 457:480–4. doi: 10.1038/nature07540, PMID: 19043404 PMC2677729

[17] Li T Lu X Yang X. Evaluation of clinical safety and beneficial effects of Stachyose-enriched alpha-Galacto-oligosaccharides on gut microbiota and bowel function in humans. Food Funct. (2017) 8:262–9. doi: 10.1039/c6fo01290f, PMID: 28001151

[18] Li T Lu X Yang X. Stachyose-enriched alpha-Galacto-oligosaccharides regulate gut microbiota and relieve constipation in mice. J Agric Food Chem. (2013) 61:11825–31. doi: 10.1021/jf404160e, PMID: 24245736

[19] Qian Y Zhao X Song JL Zhu K Sun P Li GJ. Inhibitory effects of resistant starch (Rs3) as a carrier for Stachyose on dextran sulfate sodium-induced ulcerative colitis in C57bl/6 mice. Exp Ther Med. (2013) 6:1312–6. doi: 10.3892/etm.2013.1280, PMID: 24223664 PMC3820662

[20] Chen LH Song JL Qian Y Zhao X Suo HY Li J. Increased preventive effect on Colon carcinogenesis by use of resistant starch (Rs3) as the carrier for polysaccharide of *Larimichthys Crocea* swimming bladder. Int J Mol Sci. (2014) 15:817–29. doi: 10.3390/ijms15010817, PMID: 24413751 PMC3907840

[21] Yang Y Jobin C. Novel insights into microbiome in colitis and colorectal Cancer. Curr Opin Gastroenterol. (2017) 33:422–7. doi: 10.1097/MOG.0000000000000399 28877044 PMC5826583

[22] Wei Y Liang J Su Y Wang J Amakye WK Pan J. The associations of the gut microbiome composition and short-chain fatty acid concentrations with body fat distribution in children. Clin Nutr. (2021) 40:3379–90. doi: 10.1016/j.clnu.2020.11.014, PMID: 33277072

[23] Zhao G Xie L Wu Y Wang B Teng W Sun Z. Effects of urbanization and lifestyle habits on the intestinal microbiota of adolescents in eastern China. Front Microbiol. (2023) 14:989303. doi: 10.3389/fmicb.2023.989303, PMID: 37378282 PMC10291051

[24] Liu W Li X Zhao Z Pi X Meng Y Fei D. Effect of Chitooligosaccharides on human gut microbiota and Antiglycation. Carbohydr Polym. (2020) 242:116413. doi: 10.1016/j.carbpol.2020.116413, PMID: 32564858

[25] Ye X Pi X Zheng W Cen Y Ni J Xu L. The methanol extract of Polygonatum Odoratum ameliorates colitis by improving intestinal short-chain fatty acids and gas production to regulate microbiota Dysbiosis in mice. Front Nutr. (2022) 9:899421. doi: 10.3389/fnut.2022.899421, PMID: 35634366 PMC9133717

[26] Lynch SV Pedersen O. The human intestinal microbiome in health and disease. N Engl J Med. (2016) 375:2369–79. doi: 10.1056/NEJMra1600266 27974040

[27] Hussain T Murtaza G Kalhoro DH Kalhoro MS Metwally E Chughtai MI. Relationship between gut microbiota and host-metabolism: emphasis on hormones related to reproductive function. Anim Nutr. (2021) 7:1–10. doi: 10.1016/j.aninu.2020.11.005, PMID: 33997325 PMC8110851

[28] Wikoff WR Anfora AT Liu J Schultz PG Lesley SA Peters EC. Metabolomics analysis reveals large effects of gut microflora on mammalian blood metabolites. Proc Natl Acad Sci USA. (2009) 106:3698–703. doi: 10.1073/pnas.0812874106, PMID: 19234110 PMC2656143

[29] Clemente JC Ursell LK Parfrey LW Knight R. The impact of the gut microbiota on human health: an integrative view. Cell. (2012) 148:1258–70. doi: 10.1016/j.cell.2012.01.035, PMID: 22424233 PMC5050011

[30] Zhernakova A Kurilshikov A Bonder MJ Tigchelaar EF Schirmer M Vatanen T. Population-based metagenomics analysis reveals markers for gut microbiome composition and diversity. Science. (2016) 352:565–9. doi: 10.1126/science.aad3369, PMID: 27126040 PMC5240844

[31] Lemons JMS Liu L. Chewing the fat with microbes: lipid crosstalk in the gut. Nutrients. (2022) 14:573. doi: 10.3390/nu14030573, PMID: 35276931 PMC8840455

[32] Baltazar-Diaz TA Gonzalez-Hernandez LA Aldana-Ledesma JM Pena-Rodriguez M Vega-Magana AN Zepeda-Morales ASM. Escherichia/Shigella, Scfas, and metabolic pathways-the triad that orchestrates intestinal Dysbiosis in patients with decompensated alcoholic cirrhosis from Western Mexico. Microorganisms. (2022) 10:1231. doi: 10.3390/microorganisms10061231, PMID: 35744749 PMC9229093

[33] Little AS Younker IT Schechter MS Bernardino PN Meheust R Stemczynski J. Dietary-and host-derived metabolites are used by diverse gut Bacteria for anaerobic respiration. Nat Microbiol. (2024) 9:55–69. doi: 10.1038/s41564-023-01560-2, PMID: 38177297 PMC11055453

[34] Natividad JM Lamas B Pham HP Michel ML Rainteau D Bridonneau C. *Bilophila Wadsworthia* aggravates high fat diet induced metabolic dysfunctions in mice. Nat Commun. (2018) 9:2802. doi: 10.1038/s41467-018-05249-7, PMID: 30022049 PMC6052103

[35] Wang K Liao M Zhou N Bao L Ma K Zheng Z. *Parabacteroides Distasonis* alleviates obesity and metabolic dysfunctions via production of succinate and secondary bile acids. Cell Rep. (2019) 26:222–235.e5. doi: 10.1016/j.celrep.2018.12.028, PMID: 30605678

[36] Rodriguez-Castano GP Rey FE Caro-Quintero A Acosta-Gonzalez A. Gut-derived Flavonifractor species variants are differentially enriched during in vitro incubation with quercetin. PLoS One. (2020) 15:e0227724. doi: 10.1371/journal.pone.0227724, PMID: 33264299 PMC7710108

[37] Kim CH. Complex regulatory effects of gut microbial short-chain fatty acids on immune tolerance and autoimmunity. Cell Mol Immunol. (2023) 20:341–50. doi: 10.1038/s41423-023-00987-1, PMID: 36854801 PMC10066346

[38] Liu J Tan Y Cheng H Zhang D Feng W Peng C. Functions of gut microbiota metabolites, current status and future perspectives. Aging Dis. (2022) 13:1106–26. doi: 10.14336/AD.2022.0104, PMID: 35855347 PMC9286904

[39] Zhang D Jian YP Zhang YN Li Y Gu LT Sun HH. Short-chain fatty acids in diseases. Cell Commun Signal. (2023) 21:212. doi: 10.1186/s12964-023-01219-9 37596634 PMC10436623

[40] Rahman MN Diantini A Fattah M Barliana MI Wijaya A. A highly sensitive, simple, and fast gas chromatography-mass spectrometry method for the quantification of serum short-chain fatty acids and their potential features in central obesity. Anal Bioanal Chem. (2021) 413:6837–44. doi: 10.1007/s00216-021-03639-3, PMID: 34533599

[41] Chen YT Zeng Y Li J Zhao XY Yi Y Gou M. Novel syntrophic Isovalerate-degrading Bacteria and their energetic cooperation with methanogens in methanogenic Chemostats. Environ Sci Technol. (2020) 54:9618–28. doi: 10.1021/acs.est.0c01840, PMID: 32667198

[42] Diether NE Willing BP. Microbial fermentation of dietary protein: an important factor in diet(−)microbe(−)host interaction. Microorganisms. (2019) 7:19. doi: 10.3390/microorganisms7010019, PMID: 30642098 PMC6352118

[43] Valdes DS So D Gill PA Kellow NJ. Effect of dietary acetic acid supplementation on plasma glucose, lipid profiles, and body mass index in human adults: a systematic review and Meta-analysis. J Acad Nutr Diet. (2021) 121:895–914. doi: 10.1016/j.jand.2020.12.002, PMID: 33436350

[44] Sadeghpour Heravi F Hu H. Bifidobacterium: host-microbiome interaction and mechanism of action in preventing common gut-microbiota-associated complications in preterm infants: a narrative review. Nutrients. (2023) 15:709. doi: 10.3390/nu15030709, PMID: 36771414 PMC9919561

[45] Kang A Kwak MJ Lee DJ Lee JJ Kim MK Song M. Dietary supplementation with probiotics promotes weight loss by reshaping the gut microbiome and energy metabolism in obese dogs. Microbiol Spectr. (2024) 12:e0255223. doi: 10.1128/spectrum.02552-23, PMID: 38270436 PMC10913549

[46] Yu X Gurry T Nguyen LTT Richardson HS Alm EJ. Prebiotics and community composition influence gas production of the human gut microbiota. MBio. (2020) 11:e00217-20. doi: 10.1128/mBio.00217-20 32900799 PMC7482059

[47] Braccia DJ Jiang X Pop M Hall AB. The capacity to produce hydrogen sulfide (H (2)S) via cysteine degradation is ubiquitous in the human gut microbiome. Front Microbiol. (2021) 12:705583. doi: 10.3389/fmicb.2021.705583, PMID: 34745023 PMC8564485

[48] Fu H Chen Z Teng W Du Z Zhang Y Ye X. Effects of Fructooligosaccharides and Saccharomyces Boulardii on the compositional structure and metabolism of gut microbiota in students. Microbiol Res. (2024) 285:127741. doi: 10.1016/j.micres.2024.127741 38761487

[49] Du Z Li J Li W Fu H Ding J Ren G. Effects of prebiotics on the gut microbiota in vitro associated with functional diarrhea in children. Front Microbiol. (2023) 14:1233840. doi: 10.3389/fmicb.2023.1233840, PMID: 37720150 PMC10502507

[50] Hylemon PB Harris SC Ridlon JM. Metabolism of hydrogen gases and bile acids in the gut microbiome. FEBS Lett. (2018) 592:2070–82. doi: 10.1002/1873-3468.13064, PMID: 29683480

[51] Pi XE Fu H Yang XX Yu ZC Teng WL Zhang Y. Bacterial, short-chain fatty acid and gas profiles of partially hydrolyzed guar gum in vitro fermentation by human fecal microbiota. Food Chem. (2024) 430:137006. doi: 10.1016/j.foodchem.2023.137006, PMID: 37541036

[52] Cai J Chen Z Wu W Lin Q Liang Y. High animal protein diet and gut microbiota in human health. Crit Rev Food Sci Nutr. (2022) 62:6225–37. doi: 10.1080/10408398.2021.1898336 33724115

[53] Fu Q Zhou S Yu M Lu Y He G Huang X. *Portulaca Oleracea* polysaccharides modulate intestinal microflora in aged rats in vitro. Front Microbiol. (2022) 13:841397. doi: 10.3389/fmicb.2022.841397, PMID: 35308364 PMC8931684

[54] Donati Zeppa S Natalucci V Agostini D Vallorani L Amatori S Sisti D. Changes in gut microbiota composition after 12 weeks of a home-based lifestyle intervention in breast Cancer survivors during the Covid-19 lockdown. Front Oncol. (2023) 13:1225645. doi: 10.3389/fonc.2023.1225645, PMID: 37727203 PMC10505708

[55] Raimondi S Calvini R Candeliere F Leonardi A Ulrici A Rossi M. Multivariate analysis in microbiome description: correlation of human gut protein degraders, metabolites, and predicted metabolic functions. Front Microbiol. (2021) 12:723479. doi: 10.3389/fmicb.2021.723479, PMID: 34603248 PMC8484906

